# Construction of a high-density bin-map and identification of fruit quality-related quantitative trait loci and functional genes in pear

**DOI:** 10.1093/hr/uhac141

**Published:** 2022-06-23

**Authors:** Meng-Fan Qin, Lei-Ting Li, Jugpreet Singh, Man-Yi Sun, Bing Bai, Si-Wei Li, Jiang-Ping Ni, Jia-Ying Zhang, Xun Zhang, Wei-Lin Wei, Ming-Yue Zhang, Jia-Ming Li, Kai-Jie Qi, Shao-Ling Zhang, Awais Khan, Jun Wu

**Affiliations:** College of Horticulture, State Key Laboratory of Crop Genetics and Germplasm Enhancement, Nanjing Agricultural University, Nanjing 210095, China; College of Horticulture, State Key Laboratory of Crop Genetics and Germplasm Enhancement, Nanjing Agricultural University, Nanjing 210095, China; Plant Pathology and Plant-Microbe Section, Cornell University, Geneva, NY 14456, USA; College of Horticulture, State Key Laboratory of Crop Genetics and Germplasm Enhancement, Nanjing Agricultural University, Nanjing 210095, China; College of Horticulture, State Key Laboratory of Crop Genetics and Germplasm Enhancement, Nanjing Agricultural University, Nanjing 210095, China; College of Horticulture, State Key Laboratory of Crop Genetics and Germplasm Enhancement, Nanjing Agricultural University, Nanjing 210095, China; College of Horticulture, State Key Laboratory of Crop Genetics and Germplasm Enhancement, Nanjing Agricultural University, Nanjing 210095, China; College of Horticulture, State Key Laboratory of Crop Genetics and Germplasm Enhancement, Nanjing Agricultural University, Nanjing 210095, China; College of Horticulture, State Key Laboratory of Crop Genetics and Germplasm Enhancement, Nanjing Agricultural University, Nanjing 210095, China; College of Horticulture, State Key Laboratory of Crop Genetics and Germplasm Enhancement, Nanjing Agricultural University, Nanjing 210095, China; College of Horticulture, State Key Laboratory of Crop Genetics and Germplasm Enhancement, Nanjing Agricultural University, Nanjing 210095, China; College of Horticulture, State Key Laboratory of Crop Genetics and Germplasm Enhancement, Nanjing Agricultural University, Nanjing 210095, China; College of Horticulture, State Key Laboratory of Crop Genetics and Germplasm Enhancement, Nanjing Agricultural University, Nanjing 210095, China; College of Horticulture, State Key Laboratory of Crop Genetics and Germplasm Enhancement, Nanjing Agricultural University, Nanjing 210095, China; Plant Pathology and Plant-Microbe Section, Cornell University, Geneva, NY 14456, USA; College of Horticulture, State Key Laboratory of Crop Genetics and Germplasm Enhancement, Nanjing Agricultural University, Nanjing 210095, China

## Abstract

Pear (*Pyrus* spp.) is one of the most common fruit crops grown in temperate regions worldwide. Genetic enhancement of fruit quality is a fundamental goal of pear breeding programs. The genetic control of pear fruit quality traits is highly quantitative, and development of high-density genetic maps can facilitate fine-mapping of quantitative trait loci (QTLs) and gene identification. Bin-mapping is a powerful method of constructing high-resolution genetic maps from large-scale genotyping datasets. We performed whole-genome sequencing of pear cultivars ‘Niitaka’ and ‘Hongxiangsu’ and their 176 *F*_1_ progeny to identify genome-wide single-nucleotide polymorphism (SNP) markers for constructing a high-density bin-map of pear. This analysis yielded a total of 1.93 million SNPs and a genetic bin-map of 3190 markers spanning 1358.5 cM, with an average adjacent interval of 0.43 cM. This bin-map, along with other high-density genetic maps in pear, improved the reference genome assembly from 75.5 to 83.7% by re-anchoring the scaffolds. A quantitative genetic analysis identified 148 QTLs for 18 fruit-related traits; among them, QTLs for stone cell content, several key monosaccharides, and fruit pulp acids were identified for the first time in pear. A gene expression analysis of six pear cultivars identified 399 candidates in the identified QTL regions, which showed expression specific to fruit developmental stages in pear. Finally, we confirmed the function of *PbrtMT1,* a tonoplast monosaccharide transporter-related gene responsible for the enhancement of fructose accumulation in pear fruit on linkage group 16, in a transient transformation experiment. This study provides genomic and genetic resources as well as potential candidate genes for fruit quality improvement in pear.

## Introduction

Pear (*Pyrus* spp.) is an important temperate fruit crop with a planting area of >1.38 million hectares and 23.9 million tons of global production in 2019 (www.fao.org, 9 July 2019). Although pear cultivation can be traced back around 3000 years [1], pear fruit quality traits, such as the content of stone cells and flesh flavor, still require significant improvement to satisfy consumer preferences. Understanding the molecular mechanism of genes controlling fruit quality is key for developing pear cultivars with improved fruit quality. However, it is challenging to identify genes controlling genetically complex fruit quality traits in pear due to its perennial nature, long juvenile period, and high genome heterozygosity [2].

Marker-assisted quantitative trait locus (QTL) studies have used genetic linkage maps to dissect the genetic basis of complex traits in several fruit crops [2–4]. The first genetic map of pear, developed from 82 progeny of cultivars ‘Kinchaku’ and ‘Kosui’, was constructed using random amplified polymorphic DNA (RAPD) markers [5]. In this case, the use of RAPD markers led to a separate map for each of the parents, which had unequal numbers of linkage groups. Poor reproducibility and inability to distinguish between homo- and heterozygosity made this method unable to capture complete genetic information in pear. Later studies used codominant simple sequence repeat (SSR) markers for genetic map construction [6–10], but the resulting map density was still too limited for fine-mapping traits of interest in pear.

With the availability of draft pear genome assemblies and genome re-sequencing data based on next-generation sequencing platforms [11, 12], it is now possible to develop genetic markers with uniform distribution and high density across the genome. These markers can improve genetic resolution to fine map quantitative traits and facilitate map-based gene cloning in pear. For example, a high-density single-nucleotide polymorphism (SNP)-based map was used to identify and fine map a red-skin-related QTL on linkage group (LG) 5, leading to the successful cloning of the *PyMYB114* gene, which regulates anthocyanin biosynthesis [13]. However, the tools and approaches required to develop genetic maps from millions of markers from genome re-sequencing projects are not available, and some data reduction approaches are useful to obtain the most informative markers for further analysis.

Bin-mapping is a concept proposed by Vision *et al*. [14] to identify intervals in a mapping population in which no recombination event or breakpoint occurs. In combination with whole-genome sequencing approaches, the bin-mapping strategy helps to construct highly dense genome-wide linkage maps by capturing rare recombination events in segregating populations. Therefore, these integrated approaches have been used in several species, e.g. maize [15, 16], melon [17, 18], and apple [19], to construct high-density genetic maps. In contrast to annual crop plants such as maize, segregating populations of perennial fruit plants are relatively small. Hence, bin-mapping in fruit crops is focused on reducing redundant genetic markers [17, 19] rather than selecting a smaller set of individuals where no more recombination events occur [20, 21]. For example, the ‘focal point’ (FP) method led to the clustering of up to 11 SNPs over a short genetic region to create bin markers in apples [19], resulting in the successful construction of a high-density genetic map of 15 417 SNPs that spanned a distance of 1276 cM. In this case, the bin-mapping approach was highly suited to reducing the sequencing datasets by keeping the most informative markers for high-density genetic map construction and QTL identification.

The current draft genome assembly of the Chinese pear (*Pyrus bretschneideri*) ‘Dangshansuli’ is anchored by a 2005-SNP high-density genetic map, and has only 75.5% of the scaffolds assigned to 17 pear chromosomes [11]. A high-density genetic map with 2388 SNPs from the 200-K SNP array of pear has significantly improved the pear genome assembly from 75.5 to 81.4% [22]. In this study, we report the effectiveness of the bin-mapping approach in improving the genome assembly and genetically mapping 18 fruit-related traits in pear. We further integrated gene expression and functional analysis to identify and validate candidate genes regulating sugar accumulation in pear fruits.

## Results

### DNA re-sequencing and SNP identification

**Figure 1:**
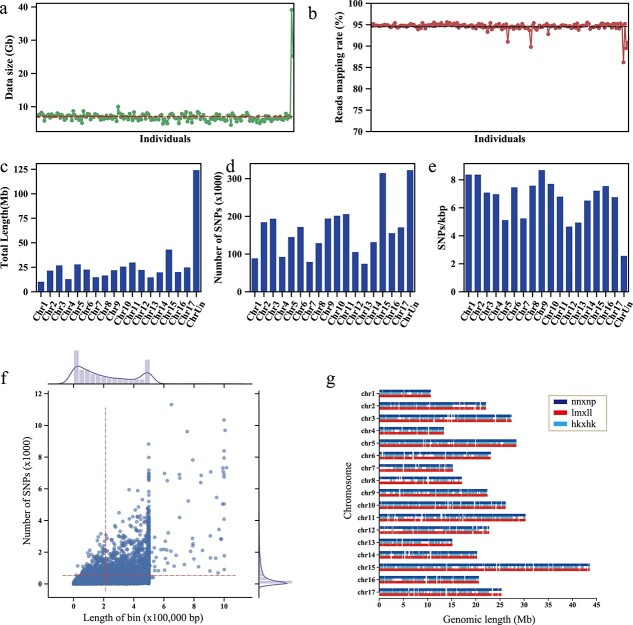
Distribution of SNP and bin markers. **a** Sequencing data size of each individual. ‘Niitaka’ and ‘Hongxiangsu’ are listed in the end. **b** Read mapping rate of each individual. **c** Total length of each pseudo-chromosome. **d** Number of SNPs in each chromosome. **e** Density of SNPs in each pseudo-chromosome. ‘ChrUn’ in **c**, **d** and **e** indicates the scaffolds that were not assembled into the pear reference genome. **f** Scatter plot of bin markers. The *x*-axis indicates the length of bin markers and the *y*-axis indicates the number of SNPs per bin. Red dashed lines indicate average marker length or number of SNPs. Subplots on the top and right side are Histplot distributions of bin length and the number of SNPs in each bin marker. **g** Distribution of three types of bin marker in each pseudo-chromosome of the pear reference genome.

Parent cultivars ‘Niitaka’ and ‘Hongxiangsu’ were sequenced at a depth of 25.3 and 39.1 Gb, respectively (Fig. 1a). A total of 1.2 terabase pairs of sequencing data was obtained (~2271-fold of the pear genome) for 176 progeny individuals from the ‘Niitaka’ × ‘Hongxiangsu’ cross, with an average of 7.1 gigabase pairs per sample. This corresponds to ~13.9-fold coverage of the sequenced pear genome for each of the progeny. Alignment of cleaned data to the Asian pear ‘Dangshansuli’ reference genome [11] produced an average mapping rate of 94.4% (Fig. 1b). Variant calling identified a total of 2 983 030 high-quality SNPs (Fig. 1c–e). SNPs were classified in three groups: hkxhk (441 192 SNPs), lmxll (1 296 204 SNPs), and nnxnp (1 245 634 SNPs). Binmarker v2.3 resulted in a total of 5515 bin markers, including 1980, 1534, and 2001 in nnxnp, hkxhk, and lmxll, respectively (Supplementary Data Table S1, Fig. 1g). Bin length averaged 213 203 bp, ranging up to 1 016 560 bp (Fig. 1f), and on average there were 541 SNPs per bin, ranging up to 11 316 (Fig. 1f).

### Construction of genetic linkage maps

Maternal and paternal maps were constructed using all 5515 bin markers. After grouping, a total of 2746 markers (1941 nnxnp and 805 hkxhk) in the paternal and 2262 markers (1400 lmxll and 862 hkxhk) in the maternal were obtained. The resulting paternal nh-map consisted of 17 LGs and spanned 1113.3 cM, with an average adjacent interval of 0.57 cM. The maternal lh-map also consisted of 17 LGs and spanned 1179.3 cM, with an average bin marker interval of 0.52 cM (Supplementary Data Table S2).

**Figure 2:**
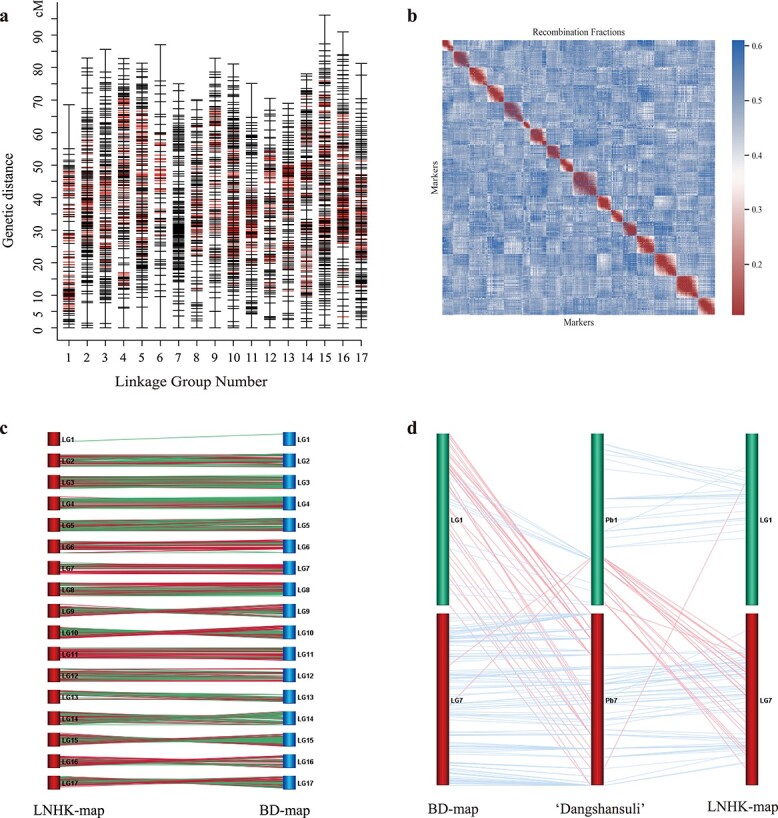
Construction of genetic map and collinearity analysis of the genetic maps and pear genome ‘Dangshansuli’. (A) Plot of marker distribution of consensus map of LNHK-map. Red bars represent the bridge marker hkxhk. (B) Recombination fractions between each pair of bins. Red color in the diagonal indicates a high recombination rate. (C) Collinearity analysis between BD-map and LNHK-map. Each line represents an SBP. A green line indicates that the SNP was exactly located in the physical position of the bin marker, and a red line indicates that an SNP and a bin marker were located on the same scaffold, but outside the bin edges. (D) Collinearity alignments plot of LG1 and LG7 from the genetic maps and chromosomes 1 and 7 of the ‘Dangshansuli’ genome.

A comparison of markers between the paternal and maternal maps identified 717 shared hkxhk loci across 16 out of 17 linkage groups (Supplementary Data Table S2). These common hkxhk loci from the corresponding linkage groups of the two parental maps were used as bridge markers to construct a consensus map in MergeMap [23]. In contrast, no common marker existed between 18 and 50 hkxhk markers in the paternal and maternal LG7 (common markers were excluded from the mapping process when constructing the parental maps due to the weak linkages using JoinMap software), and all the markers from the paternal and maternal LG7s were combined to reconstruct a new linkage group using the same parameters, named ‘LG7’. The consensus LNHK-map generated from the paternal and maternal maps consisted of 17 LGs (16 fused groups and one reconstructed group), which contained a total of 3190 markers, including 1400 (43.9%) lmxll, 887 (27.8%) nnxnp, and 903 (28.3%) hkxhk. This map spanned 1358.5 cM, with an average interval of 0.43 cM between adjacent markers (Table 1, Fig. 2A, Supplementary Data Table S3). The genetic length of the LGs ranged from 68.6 (LG1) to 96.1 cM (LG15) with an average length of 79.9 cM. LG6 contained the fewest markers (*n* = 81) and had the widest mean interval (1.09 cM) between adjacent markers. LG10 contained highest number of markers (*n* = 279) and the narrowest mean interval (0.29 cM). There were on average 188 bins per linkage group (Fig. 2A and Table 1). The total number of SNPs in the mapped bin loci for each LG ranged from 57 081 to 174 423 (LG13 and LG9, respectively), with 114 005 SNPs on average. In total, LNHK-map contained 1 938 092 SNPs in 3190 bins that were distributed across 17 linkage groups. Pairwise recombination fractions among all 17 linkage groups were located on the diagonal of the heat map, indicating high quality of the LNHK-map (Fig. 2B).

**Table 1:** Information on 3190 bin markers and 1.93 Mb SNP markers mapped on the 17 linkage groups of pear genetic map LNHK-map.

Linkage group	Number of markers	Length (cM)	Average interval (cM)	Number of SNPs	Number of co-anchored markers	Number of misalignments	Number of new anchored markers	Percentage of new anchored markers	Percentage of collinearity^a^
1	127	68.6	0.54	85 736	23	23	81	63.8	50.0
2	190	82.9	0.44	145 354	84	27	79	41.6	75.7
3	207	85.6	0.42	148 991	130	25	52	25.1	83.9
4	197	82.8	0.42	106 518	69	29	99	50.3	70.4
5	225	81.4	0.36	80 388	119	23	83	36.9	83.8
6	81	87.0	1.09	38 453	49	10	22	27.2	83.1
7	189	75.0	0.40	85 465	55	38	96	50.8	59.1
8	160	70.1	0.44	129 736	84	14	62	38.8	85.7
9	152	82.9	0.55	174 423	97	23	32	21.1	80.8
10	279	81.1	0.29	155 804	134	32	113	40.5	80.7
11	167	75.1	0.45	96 570	96	18	53	31.7	84.2
12	138	70.5	0.51	82 716	89	8	41	29.7	91.8
13	166	69.0	0.42	57 081	35	22	109	65.7	61.4
14	203	78.1	0.39	112 610	97	25	81	39.9	79.5
15	258	96.1	0.37	166 353	144	25	89	34.5	85.2
16	252	91.0	0.36	152 038	117	24	111	44.1	83.0
17	199	81.2	0.41	119 856	101	17	81	40.7	85.6
Average	188	79.9	0.43	114 005	90	23	76		
Total	3190	1358.5	—	1 938 092	1523	383	1284	40.3	79.9

a Collinearity was calculated as the percentage of markers that were co-anchored (number of co-anchored markers divided by total number of co-anchored and misaligned markers).

### Collinearity analysis and improvement of the pear reference genome assembly

We performed collinearity analysis using LNHK-map with another SNP-based high-density genetic map, BD-map [2], to identify SNP–bin pairs (SBPs) located on the same scaffolds in the pear genome (Fig. 2C). These two maps shared 7899 SBPs, of which 7289 (92.3%) were mapped on the same linkage groups. Surprisingly, there was only one SBP detected on LG1; to determine why this was the case, we further performed collinearity analysis of LNHK-map and BD-map with the reference genome of pear cultivar ‘Dangshansuli’ (Supplementary Data Fig. S1). Compared with the reference genome, we divided the SNPs and bin markers from our maps into three marker types: (i) markers mapped to the corresponding chromosome (co-anchored); (ii) markers mapped to a chromosome other than the corresponding genome reference chromosome (misaligned); and (iii) markers located in the unanchored scaffolds of the pear genome (unassembled).

Comparing LNHK-map with the reference showed that, on average, 79.9% of assembled markers (ranging from 50.0% in LG1 to 91.8% in LG12) were mapped to the corresponding linkage groups (Table 1). In particular, the collinearity of LG7 was <60%, because most of the misaligned markers (24 of 38) in LG7 were from chromosome 1 (Chr1) of the ‘Dangshansuli’ genome (Fig. 2D, Table 1), which has high homology with chromosome Chr7 as a result of an ancient whole-genome duplication event [11]. Comparing BD-map with the reference pear genome ‘Dangshansuli’ (Supplementary Data Table S6), the average percentage of co-anchored markers was 89.2%, ranging from 26.0% in LG1 to 98.1% in LG13. Since the collinearity of LG1 (in both LNHK-map and BD-map) with Chr1 of the reference genome was quite low, we considered that this is the most likely reason why there was only one SBP identified on LG1. Further comparison of the physical distance of the three maps (Fig. 2d) showed that BD-map LG1 corresponded to the bottom of ‘Dangshansuli’ Chr1, while LNHK-map LG1 corresponded to the top. Additionally, 33 out of 50 SNPs in BD-map LG1 corresponded to Chr7 in the ‘Dangshansuli’ genome, and were identified in LG7 of LNHK-map through the SBPs. These results suggest that there are assembly errors in Chr1 and Chr7 of the current pear genome assembly ‘Dangshansuli’-v1.

**Figure3:**
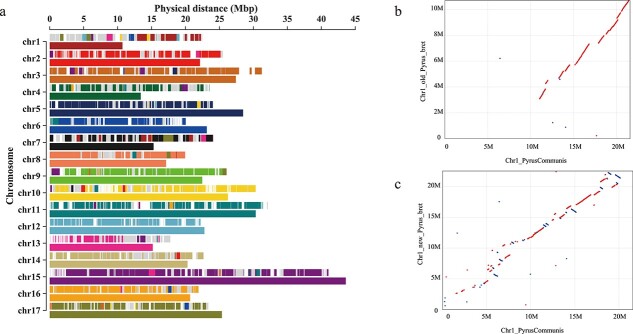
Genome assembly of ‘Dangshansuli’ and collinearity comparison of Chr1 from the new genome assembly and BartlettDHv2.0 (*Pyrus communis* L.). (A) Comparison of ‘Dangshansuli’ genome (bottom) and new assembled genome alignment (top) according to the collinearity of BD-map and LNHK-map. Different colored boxes represent scaffolds originating from different chromosomes of the ‘Dangshansuli’ genome. Gray boxes represent unassembled scaffolds in the ‘Dangshansuli’ genome. (B) Dot plot of chromosome 1 ‘Dangshansuli’-v1 to BartlettDHv2.0. (C) Dot plot of chromosome 1 of new ‘Dangshansuli’-v2 to BartlettDHv2.0.

Based on the collinearity of BD-map and LNHK-map, we re-anchored the reference genome, ‘Dangshansuli’-v2, which had assembled a total of 1316 scaffolds (480 more than the first version). The total length of ‘Dangshansuli’-v2 was 428.14 Mb, accounting for 83.7% of the pear genome and representing 8.1% assembly improvement compared with the first version (Supplementary Data Table S7). Specifically, the length of Chr1 increased from 10.7 to 20.8 Mb (Fig. 3a). The assemblies of Chr4 and Chr7 were also greatly improved from 13.4 to 23.4 Mb of Chr4 and from 15.3 to 30.0 Mb of Chr7. However, the lengths of Chr5, Chr6, Chr12, Chr15, and Chr17 were slightly reduced (Fig. 3A).

To verify the accuracy of the new assembly, we compared Chr1 of the new European pear genome BartlettDH-v2.0 assembly (*Pyrus communis*) with both ‘Dangshansuli’-v1 and ‘Dangshansuli’-v2. As shown in Fig. 3B and C, ‘Dangshansuli’-v1 Chr1 corresponded to the part of BartlettDH Chr1 from ~11 Mb to the end, with the top 10 Mb missing. The top 10 Mb was no longer missing in our new ‘Dangshansuli’-v2 assembly and showed collinearity with BartlettDH-v2.0. This result demonstrates that ‘Dangshansuli’-v2, our new assembly based on the collinearity of SBPs in BD-map and LNHK-map, has increased assembly length and accuracy compared with ‘Dangshansuli’-v1.

### Phenotyping of fruit-related traits

**Figure 4:**
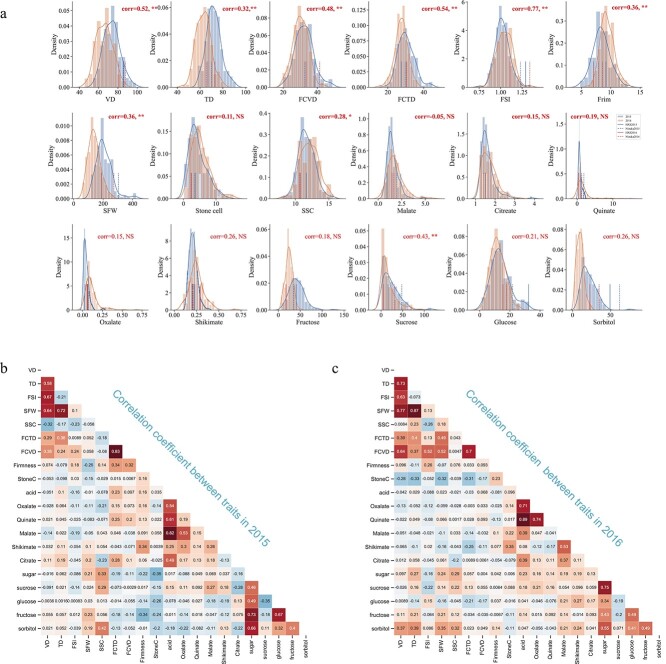
Distribution and correlation of phenotypic data of fruit related traits. **a** Phenotypic data distribution pattern of 18 fruit-related traits in 2015 (blue) and 2016 (orange). The *x*-axis of each subplot indicates the range of variation and the *y*-axis indicates frequency density. Correlation coefficients between the two years were computed by scipy.stats.pearsonr(x,y), ‘^**^’ represents a significance level of 0.01, and ‘^*^’ for 0.05. NS, not significant. **b**, **c** Heat maps of correlation analysis among 18 traits in years 2015 (left) and 2016 (right).

The continuous variation of all 18 fruit quality traits from the ‘Niitaka’ × ‘Hongxiangsu’ segregating population demonstrates the quantitative nature of these traits (Fig. 4a, Table 2). Shapiro–Wilk analysis [24] (Fig. 4a) showed that 11 traits had a normal distribution; these comprised fruit vertical diameter (VD, fruit length), fruit transverse diameter (TD, fruit width), fruit shape index (FSI), fruit core vertical diameter (FCVD), fruit core transverse diameter (FCTD), single fruit weight (SFW), soluble solids content (SSC), flesh firmness, and the glucose, fructose, and sorbitol contents. The remaining seven traits, stone cell content (SCC), sucrose, malate, citrate, oxalate, shikimate, and quinate levels, had positive-skewed distributions.

**Table 2:** Phenotypic distribution of 18 fruit-related traits in 176 *F*_1_ progenies from hybridization of pear ‘Niitaka’ and ‘Hongxiangsu’.

Trait	Year	Minimum	Maximum	Mean	Range	Standard deviation	Kurtosis^a^	Skewness^a^	*P*-value^b^
Vertical diameter (mm)	2015	54.03	98.82	73.61	44.80	8.55	−0.06	0.29	0.44
	2016	45.25	87.51	65.90	42.26	8.41	−0.38	0.14	0.55
Transverse diameter (mm)	2015	58.04	91.37	72.06	33.33	6.42	0.26	0.40	0.07
	2016	44.53	80.89	63.01	36.36	6.43	0.06	0.19	0.63
Fruit shape index	2015	0.74	1.35	1.02	0.61	0.09	1.49	0.47	0.01
	2016	0.77	1.36	1.05	0.59	0.09	0.41	0.11	0.58
Single fruit weight (g)	2015	116.67	424.67	206.96	308.00	54.78	2.78	1.22	0.00
	2016	58.50	267.83	140.82	209.33	41.12	0.12	0.51	0.02
Soluble solids content (%)	2015	8.00	15.70	11.66	7.70	1.22	0.63	0.31	0.26
	2016	8.17	15.33	12.07	7.17	1.36	−0.10	−0.02	0.53
Fruit core transverse diameter (mm)	2015	18.57	44.84	30.22	26.27	4.48	0.70	0.57	0.01
	2016	15.13	36.62	27.53	21.49	3.60	0.60	−0.29	0.40
Fruit core vertical diameter (mm)	2015	19.95	48.90	32.52	28.94	5.37	0.52	0.44	0.03
	2016	19.58	43.78	30.18	24.20	4.48	0.08	0.04	0.72
Firmness (kg cm^−2^)	2015	4.77	12.97	8.41	8.20	1.48	0.42	0.24	0.85
	2016	6.07	12.80	9.40	6.73	1.18	0.36	0.40	0.08
Stone cell content (g kg^−1^)	2015	0.14	13.73	4.41	13.59	2.71	0.56	0.93	0.00
	2016	0.36	15.41	5.25	15.05	2.84	1.49	1.07	0.00
Oxalate (mg g^−1^)	2015	0.00	0.27	0.04	0.26	0.04	14.96	3.06	0.00
	2016	0.01	0.69	0.11	0.67	0.09	12.24	2.97	0.00
Quinate (mg g^−1^)	2015	0.18	2.23	0.81	2.05	0.47	0.97	1.26	0.00
	2016	0.19	14.94	1.64	14.75	2.11	16.41	3.79	0.00
Malate (mg g^−1^)	2015	0.59	4.82	1.52	4.23	0.58	6.58	1.83	0.00
	2016	0.30	5.73	1.83	5.44	0.87	3.57	1.46	0.00
Shikimate (mg g^−1^)	2015	0.14	0.39	0.21	0.25	0.04	1.29	0.88	0.00
	2016	0.09	0.69	0.25	0.59	0.08	3.96	1.10	0.00
Citrate (mg g^−1^)	2015	1.23	3.88	1.63	2.65	0.36	11.26	2.70	0.00
	2016	1.18	3.26	1.71	2.08	0.39	1.52	1.12	0.00
Sucrose (mg·g^−1^)	2015	1.37	116.98	23.95	115.61	20.26	4.76	1.75	0.00
	2016	0.55	94.58	17.26	94.03	17.87	3.82	1.89	0.00
Glucose (mg g^−1^)	2015	1.19	32.79	13.86	31.60	6.47	0.52	0.71	0.00
	2016	0.13	26.32	11.94	26.19	4.85	0.05	0.38	0.21
Fructose (mg g^−1^)	2015	7.52	127.07	42.46	119.55	17.40	3.17	1.02	0.00
	2016	5.64	49.89	24.45	44.25	8.28	−0.05	0.09	0.53
Sorbitol (mg g^−1^)	2015	6.92	76.65	21.76	69.72	9.83	5.33	1.49	0.00
	2016	1.64	25.26	10.17	23.61	4.71	0.31	0.61	0.00

a Kurtosis and skewness were calculated by SPSS 20.0 to fit the type of distribution.

b The *P*-value computed by Scipy.stats.shapiro(x) was used to test the significance of normal distribution; *P* > .05 indicates the data are normal distributed.

A correlation analysis for each trait between the two years revealed different correlation trends (Fig. 4a). The correlation coefficients ranged from 0.24 for quinate to 0.73 for FSI; VD, TD, FSI, FCVD, FCTD, SFW, SSC, firmness, and sucrose, fructose, glucose, sorbitol, and shikimate levels had significant positive correlations (*P* = 0.01) between 2015 and 2016, while the other traits were not significantly correlated (Fig. 4a). In addition, traits VD, TD, FSI, SFW, FCTD, FCVD, and SCC showed high heritability (0.4–0.8) and genetic variance in at least one year, and sugar content traits showed slightly lower heritability than the outer appearance traits (0.10–0.45). Acid traits showed the lowest heritability (0.00–0.61; Supplementary Data Table S8). A correlation analysis of the 18 fruit-related traits in separate years showed significant correlations between SFW, TD, VD, FCTD, FCVD and FSI (Fig. 4b and c); SSC had a significant positive correlation with firmness, but was negatively correlated with SFW, TD, and VD; and SSC was positively correlated with sucrose and sorbitol, while exhibiting a negative correlation with citrate (Fig. 4b and c).

### Quantitative trait locus analysis

**Figure 5:**
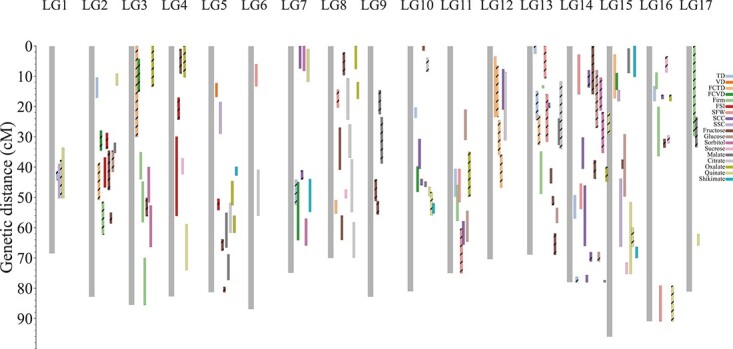
Two years’ QTL distributions of 18 fruit-related traits on LNHK-map. Gray bars represent the 17 linkage groups of the LNHK-map and colored bars represent the potential QTLs for different fruit traits. Bar length indicates the 2 LOD-drop interval of potential QTLs. Pure colored bars represent QTLs identified from the year 2015 and bars filled with slashes represent those from 2016.

Interval mapping and the Kruskal–Wallis (KW) test were used to identify putative QTLs for different fruit-related traits. We identified 148 QTLs (Fig. 5, Supplementary Data Fig. S2, Supplementary Data Table S4) using the significant logarithm of odds (LOD) threshold of 2.5. QTLs for sugar content and acidity were divided into those associated with each of the four monosaccharides and five organic acids among the fruit-related traits and were independently mapped on different LGs (Fig. 5). Although the 18 fruit-related traits were quantitatively distributed, only 12 traits of putative QTLs could be confirmed in both 2015 and 2016 within the same or adjacent regions (<10 cM). These QTLs corresponded to FSI on LG2 and LG3, SCC on LG7 and LG14, shikimate on LG7 and LG15, fructose on LG8, quinate on LG10 and LG15, SFW on LG11, FCTD on LG12, and TD on LG14. Some QTLs were identified on different LGs in the two years. For example, in 2015, QTLs for SFW were identified on LG7, LG11, LG14, and LG16, while LG11 and LG13 contained QTLs for this trait in the year 2016. The highest LOD score for SFW in 2015 was on LG14 (4.57, explaining 11.3% of the variation), and in 2016 it was on LG13 (3.93, explaining 10.5% of the variation).

QTLs were unevenly distributed across all 17 linkage groups (Fig. 5). Some linkage groups contained QTLs for several fruit traits with apparent clustering across fewer genomic regions. For example, LG14 had QTLs representing >10 traits, while LG6, LG9, and LG17 had QTLs for no more than 3 traits. In total, there were 15 QTL clusters on 10 LGs, most of which represent traits that were correlated with one other. For example, LG14 had the most QTLs (*n* = 18) for 10 fruit traits, which were distributed among three clusters. The top region of LG14 (9–21.5 cM) showed QTLs for SFW, SCC, and sugars; in the middle regions (32.5–49.7 cM) there were QTLs for SCC and sugars; and the bottom (67.0–78.1 cM) contained QTLs for SCC, malate, quinate, firmness, and TD. According to the correlation analysis (Fig. 4b and c), SCC was negatively correlated with SFW (−0.32, *P* = 0.05) and positively correlated with firmness (0.23, *P* = 0.05) and malate (0.22, *P* = 0.05). Similarly, LG13 had 14 QTLs for nine fruit traits. However, LG6 had only one QTL for citrate. Most of the QTLs for the 18 fruit-related traits had minor effects and explained 6.3–17.5% of the phenotypic variation (SFW at 45.4 cM on LG14 in 2015 and firmness at 55.1 cM on LG3 in 2015), and the average variation explained was 8.2%.

### Candidate genes in quantitative trait locus regions

The candidate genes associated with the traits were found by using the physical location on the pear reference genome corresponding to the bin marker. In order to narrow down the range of candidate genes, we only selected significant markers in the ‘1 LOD-drop off’ interval for preliminary screening of candidate genes. As a result, a total of 8241 candidate genes associated with 18 traits were identified. To further screen the candidate genes related to the studied fruit traits, we analyzed transcriptome data of six pear cultivars at developmental stages ranging from fruit setting to senescence after harvesting. Combining transcriptome profiles with the functional annotations, we were able to select 399 candidate genes for the respective QTLs (Supplementary Data Table S5). For example, among 504 candidate genes related to SCC (Supplementary Data Fig. S4a), 43 were abundantly expressed during the early fruit developmental stages. A previous study [25] showed that some of these candidate genes have important functions in regulating SCC accumulation, including transcription factors from the *MYB*, *bHLH*, and *WRKY* families, as well as *Laccase* (*LAC*) and *HCT*. Similarly, some genes are expressed specifically during later fruit developmental stages and match the pattern of sugar accumulation during fruit ripening in pear [26, 27]. For example, gene candidates for sucrose levels were highly expressed during the last three developmental stages (Supplementary Data Fig. S4b). These gene candidates for sugar content include sugar transporters, zinc finger, F-box, MYB transcription factors, bHLH (family of basic helix–loop–helix transcription factors), and E3 ubiquitins (Supplementary Data Table S5).

### Overexpression of sugar-related gene *PbrtMT1*

**Figure 6:**
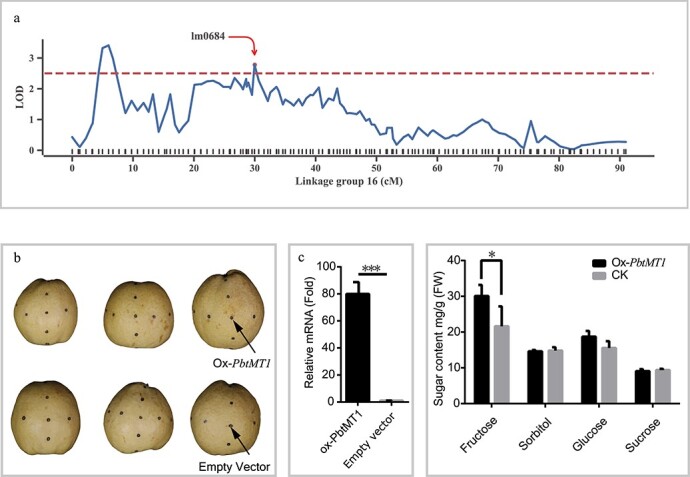
QTL mapping and transient overexpression of sugar transporter gene *PbrtMT1*. **a** LOD value of interval mapping for sucrose in the year 2016. **b** Transient injection of *PbrtMT1* construct in mature pear fruit (‘Dangshansuli’). *Ox-PbrtMT1* and empty vector were injected into several dots cross the middle line of the fruit (injection points were on both the horizontal and vertical equatorial lines). **c** Relative expression of *PbrtMT1* at the same fruit developmental stage in ‘Dangshansuli’. **d** Sugar content in the fleshy tissue around the injected region. ‘*’ represents a significant difference (<.05). ‘***’ represents a significant difference (<.005). Error bars indicate the standard error from three independent experiments.

A candidate gene for sugar content was identified that fell within a significant QTL on LG16 identified in 2016 (Fig. 6a). This gene, *PbrtMT1*, a tonoplast monosaccharide transporter [28], was identified using the bin marker lm0684 (LOD = 3.13, 31.0 cM on LG16). The genomic length of the lm0684 bin was ~477.5 kb, and *PbrtMT1* was located 159.5 kbp upstream from lm0684 on Chr6. As shown in Fig.S4a, the expression of *PbrtMT1* (*Pbr015095.1*) was relatively high in the latter three stages of fruit development, and showed similar expression trends in different pear cultivars except for ‘Nanguoli’, which was consistent with sugar accumulation trends, indicating the candidate gene might involve in the regulation of sugar content in pear fruit.

Furthermore, transient transformation in ‘Dangshansuli’ pear was performed to test the function of this candidate gene, *PbrtMT1*, in maturing pear fruits. Infection liquid with an overexpression construct and empty vector was injected into near-mature-stage pear fruits (Fig. 6b). Fruits with transient expression exhibited high levels of *PbrtMT1* compared with the control (Fig. 6c), and the injected areas showed a higher sugar content compared with the non-transgenic fruits (Fig. 6d). This indicated that *PbrtMT1* can promote higher sugar accumulation in pear fruits. In particular, fructose showed a significant difference (*P* < .05) between transgenic and non-transgenic pear fruits (Fig. 6d), consistent with its role as the main sugar component of all four monosaccharides in the selected fruit traits.

## Discussion

The cutting-edge next-generation sequencing sequencing techniques can produce thousands to millions of high-quality DNA markers. This can be computationally challenging and time-consuming for genetic linkage map construction from most common and widely used mapping software [29, 30]. The bin-mapping strategy can use most informative markers from large marker datasets to overcome these limitations, but can still capture the full recombination landscape of a mapping population. Merging consistent markers as recombinant bins eliminates the redundant loci, reducing marker placement errors and miscalculation of genetic distances during map construction. This approach has been successfully implemented in several species to generate ultra-dense genetic linkage maps [15, 31, 32]. In high-yield crops such as maize and rice, the bin-mapping strategy has been used to find the smallest set of intervals or bins by identifying progeny representative of all the recombination events from a given set of individuals [20, 21]. Fruit crops, such as apple and pear, are highly heterozygous and self-incompatible, meaning the first generation (*F*_1_) can be used to study the genetic control and lack the resolution in genetic maps. In addition, populations are generally too small to detect all of the recombination events. To address this shortcoming, a strategy similar to bin-mapping, named focal point (FP), was adopted in apple [19]. FP markers represent clusters of SNPs (up to 11) within short genomic distances to reduce the number of markers for genetic mapping. However, within a specific FP marker, different segregation events were not separated out. This means that the segregation of an FP marker could change depending on the single marker used to represent a given genetic region, and cannot perfectly represent the true locus segregation in a genotype.

We used the bin-mapping strategy to merge 100% identical genotypes within a sliding window to reduce the number of markers, but the markers generated were able to represent genome-wide recombination patterns in pear. More importantly, we found the bin-map to be more accurate than the SNP-based traditional mapping approach. For example, the collinearity analysis with the SBPs, while constructing non-reference genetic maps, showed that SNPs from the same scaffold were grouped into different linkage groups, or were in the incorrect order compared with the scaffold’s sequences. The longer distance of bin markers was helpful in correcting these errors (Fig. 2d). This was evident from the low collinearity of the LNHK-map to the pear genome for LG1 and LG7 (Table 1), but the high collinearity of the SBPs between BD-map and LNHK-map proved that some of the scaffolds placed on Chr1 actually belong to Chr7 (Fig. 3a). This has probably occurred due to high homology between Chr1 and Chr7 as they are derived from the same ancestral chromosome through a whole-genome duplication event [11]. We further improved the orientation and quality of pear genome assemblies with the genetic maps from the bin-mapping approach, as shown in Fig. 3a. Similar methods had been used in melons, where 90% of the genome scaffolds were reoriented after re-anchoring sequence reads using a dense bin-map [18]. In this study, we used the collinearity of marker orders from two separate genetic maps (‘Bayuehong’ × ‘Dangshansuli’, and ‘Niitaka’ × ‘Hongxiangsu’) to re-anchor the scaffolds of the pear genome, and generated the ‘Dangshansuli’-v2 pear genome. The scaffolds were cut by the edge of bin markers, and their position and orientation were determined by the SBPs of BD-map and LNHK-map collinearity. It was worth noting that the two genetic maps, BD-map and LNHK-map, had similar genetic backgrounds to the reference pear genome ‘Dangshansuli’ (*P. bretschneideri*). For example, in BD-map, ‘Dangshansuli’ is one of the parents of the population. As to the parents of the LNHK-map, ‘Hongxiangsu’ is a hybrid of *P. bretschneideri* × *P. sinkiangensis*, which is very close in genetic background. As shown in Fig. 1b, the average of mapping reads of progenies was ~95%, which also indicated that this population was close to the pear reference genome genetically. Collinearity analysis of the two maps ensured the quality of the genome reassembly. These results suggest that even though recombination events within a single cultivar are limited, analysis of collinearity among multi-background genetic maps could improve marker order.

This bin-map has the smallest average marker intervals (0.43 cM) compared with previous linkage maps in pear [2, 5–10, 22], making it ideal for high-resolution genetic mapping of desirable traits. We identified 180 QTLs for 18 fruit-related traits in pear. Out of them, the 14 QTLs for SCC were identified for the first time in pear. Moreover, the QTL results from this study are consistent with previous findings, where most QTLs for sugar and acid levels and firmness showed low heritability and high complexity. For example, SFW QTLs were detected on LG8 and LG13 in this study, whereas previously QTLs for SFW have been detected on LG2, LG7, LG8, LG13, and LG17 of ‘Bayuehong’ × ‘Dangshansuli’ [2]. This indicates that QTLs for SFW on LG8 and LG13 are more likely to contain genes important in SFW across multiple cultivars. Similarly, we identified SSC QTLs on LG5 and LG15, while SSC QTLs on same genomic regions on LG5 and LG10 were identified [2, 10], suggesting that conserved genes on these pear chromosomes regulate SSC. We also identified less repeatable QTLs in this study. We found QTLs for malate, one of five major acid compounds in pear fruit [33], only on LG2, LG13, LG15, and LG17 (Fig. 5), which confirmed the results of a previous study [34]. In addition, we identified several minor effect QTLs for many fruit traits, indicating their highly complex genetic regulation. Several studies have reported similar results for fruit quality traits in different species. For example, Zhang *et al*. [35] reported that QTLs for acidity explained only ~13.5% of observed phenotypic variation in apple, and QTLs of acidity and sweetness detected by Rymenants *et al*. [36] explained from 2.5 to 33.9% of the phenotypic variation (13.9% on average). Minor QTLs explaining 1.3–19% of the phenotypic variance were also reported in grapes for number of clusters, cluster weight, number of berries, and berry weight [37]. These results suggest that marker-assisted selection for fruit quality traits may not be as powerful as it could be in traits controlled by single or a few genes. However, minor-effect QTLs are useful for the application of genomic selection and predictive breeding in pear fruit quality improvement [38–40].

The results of this study also led to the identification of several candidate genes for different fruit quality traits. For example, previous studies have revealed that the formation of stone cells, a trait causing poor fruit quality, is closely related to the biosynthesis, transformation, and deposition progress of lignin in pear flesh [41]. We have identified 43 candidate genes for SCC (Supplementary Data Fig. S4b). Among these genes, *Pbr035962.1* (Supplementary Data Fig. S3) on LG12 was annotated as *laccase* (*LAC*), which was reported as necessary and non-redundant for lignin polymerization in *Arabidopsis* and *Brachypodium distachyon* [42–44]. Xue *et al*. [25] confirmed that the expression of *Pbr035962.1* (*PbrLAC2*) was positively correlated with lignin content during fruit development stages, and silencing the gene led to a slight reduction in lignin content. Another candidate gene identified on LG12 in the year 2015, *Pbr0359257.1*, is annotated as an MYB transcription factor that has been reported to have an important role in regulating lignin biosynthesis and cell wall formation in many species, such as *Zea mays* [45], *Arabidopsis thaliana* [46], and loquat [47]. Another candidate gene, a heat shock factor (*HSF*), has previously been associated with cell wall modification and lignin biosynthesis [48]. These candidate genes may play important roles in regulating lignin biosynthesis, and therefore SCC in pear. Similarly, we found annotated genes for sugar content that may have either a direct or an indirect impact on the accumulation of sugars in pear. These genes belong to electron transfer, sugar transport, MYB transcription factors, and the tricarboxylic acid (TCA) cycle and glycolysis pathways (Supplementary Data Table S5). A QTL for sucrose on LG16 contained a candidate gene, *PbtMT1* (gene ID *Pbr015095.1*) (Fig. 5), which was annotated as a ‘sugar/inositol transporter’ and was previously predicted to be a tonoplast monosaccharide transporter (*tMT*) [28]. *tMT* genes in *Arabidopsis*, *AtTMT2*, and *AtTMT3*, have been identified as fructose/H^+^ or glucose antiporters on vacuolar membranes [49, 50], which loads vacuoles with glucose and sucrose. More recently, Cheng *et al*. [26] identified *PbtMT4* as a tonoplast sugar transporter making strong contributions to sugar accumulation during pear fruit ripening. Consistent with these previous findings, we also found that the overexpression of *PbtMT1* significantly increased the accumulation of fructose, a major sugar component in fruit, in pear cultivar ‘Dangshansuli’ (Fig. 6d).

Fruit quality is particularly influenced by the environmental conditions. Our 18 fruit-related traits included outer appearance (TD, VD, FSI, SFW), core size (FCVD and FCTD), and flesh-related quality traits (firmness, SSC, SCC, sugars, and acids). Comparatively, the flesh-related traits had no significant correlation between 2015 and 2016, except for fruit firmness and content of sucrose (Fig. 4a). Two major reasons might contribute to this phenomenon: one reason might be the climatic conditions during maturity. According to statistics from the Meteorological Department, the annual precipitation in 2016 was the highest precipitation year since 1951. The high temperature and high humidity of the environment could result in lower sugar content. This is in line with the fact that the sugar content in 2016 was generally lower than in 2015. Another reason might be the maturity period; early or delayed harvest time depending on judgment and environmental change may affect the content of sugar and acidity.

In conclusion, we demonstrated the utility of whole-genome resequencing and high-density genetic bin-mapping for the identification of reliable QTLs and candidate genes in a single *F*_1_ hybrid population in pear. We believe that similar approaches can be useful in other fruit crops as well.

## Materials and methods

### Plant materials

A first-generation family of pear cultivar ‘Niitaka’ (*P. pyrifolia*; female parent) × ‘Hongxiangsu’ (*P. sinkiangensis* × *P. bretschneideri*; male parent) was created by hybridization at the Jiangpu Horticulture Experimental Station of Nanjing Agricultural University, Nanjing, Jiangsu, China in 2008. The fruits of ‘Niitaka’ and ‘Hongxiangsu’ have great phenotypic differences. For example, the fruits of ‘Niitaka’ usually have a nearly round shape, large fruit weight (average single fruit weight 450–500 g), and yellow-brown skin color, and the maturity period is mid- to late September. ‘Hongxiangsu’ fruits are generally oval or spindle-shaped, with an average weight of 200 g, and have a yellow-green background with bright red on the yang side. In addition, its fruit has an aromatic smell. Maturity is in late September. Since these two pear cultivars have such large phenotypic differences, the hybrid population of these two pear cultivars is particularly suitable for studying the genetic regularity of pear fruit traits.

A total of 700 seedlings were grown without grafting and were managed under uniform conditions. We selected 176 individuals (the ones that had stable fruit setting for the two consecutive years of the study) to construct genetic linkage maps and perform QTL mapping analysis. Three to five young leaves (not fully expanded) were randomly collected from each individual for DNA extraction and whole-genome re-sequencing. Mature fruits harvested from every plant were used for various trait measurements. Fruit maturity was judged from external and internal indicators to ensure that the fruit on a tree had reached physiological maturity, including checking the smoothness, gloss, and smell of the skin, tasting the flavor of the pulp, and observing the lignification degree of the seeds and the color of the pulp. The fruit harvest date of each plant in the hybrid population was not recorded.

### Trait phenotyping and heritability calculation

In two growing seasons (2015 and 2016), five to eight mature fruits at physiological maturity were randomly collected from the perimeter of the tree crown for trait measurement. In total, 18 fruit-related traits related to outer appearance, core size of fruit, and flesh-related quality traits were measured. For flesh-related quality traits, pulps of five to eight fruits were mixed together and divided into three replicates for trait evaluation. For the outer appearance quality traits and core size, three fruits of similar size were individually measured for TD, VD, FSI, SFW, fruit firmness, FCVD, and FCTD, and the average value was used. TD and VD were measured by digital Vernier caliper (unit: mm), FSI was calculated as VD/TD, and SFW was determined with digital scales (G&G Electronic Scale T2000, Powerhouse®) (unit: g). The size of the fruit core was measured based on FCVD and FCTD (unit: mm) using the digital Vernier caliper, and fruit firmness was measured using a hand-held penetrometer (GY-1 X, 3.5 mm). The flesh-related traits included SCC, SSC, five organic acid concentrations, and four soluble saccharide concentrations. SCC of fruit flesh was extracted using hydrochloric acid [51] after 24 hours of storage at −20°C and weighed with an electronic balance (unit: g/kg). SSC was measured by refractometer (Atago, model N-1). Measurements of soluble saccharides and organic acids were performed using a Waters 1525 HPLC system (Waters, Shanghai, China). A Hi-Plex Ca Column (7.7 mm × 300 mm, 8 μm, Agilent) and a Hi-Plex Ca Guard Column (7.7 mm × 50 mm, Agilent) were used to isolate and detect four major soluble sugars (fructose, glucose, sucrose, and sorbitol). A Zorbax SB-C18 column (4.6 mm × 250 mm, 5 μm, Aglient) was used for the detection of five organic acids (malate, quinate, oxalate, shikimate, and citrate). Detailed parameters and steps described by Yao *et al*. [52] and Liu *et al*. [53] were used for soluble sugar and organic acid detection.

To evaluate the variance and distribution patternof phenotypic data, one-way ANOVA analysis,correlation analysis, and normality analysis were performed using stats.f_oneway(x,y), stats.pearsonr(x,y), and stats.shaprio(x) from Scipy in Python. The narrow-sense heritability of each trait was estimated as ${h}^2=\frac{\upsigma_g^2}{\upsigma_g^2+{\upsigma}_e^2}$ by using the rrBLUP/R package (https://cran.r-project.org/web/packages/rrBLUP/), where ${\upsigma}_g^2$ represents the genetic variance and ${\upsigma}_e^2$ represents residual variance.

### DNA extraction and re-sequencing

Young leaves were collected from the two parent cultivars ‘Niitaka’ and ‘Hongxiangsu’ and from 176 *F*_1_ individuals. DNA extraction was performed using the Plant DNA Isolation Kit (DE-0611, http://www.foregene.com). The DNA sequencing libraries were constructed using the Truseq Nano DNA HT Sample Preparation Kit (Illumina, USA) and sequenced on the Illumina HiSeq2500 platform following the manufacturer’s instructions. Paired-end 150-bp reads were generated from the whole genomes of parents and the segregating progeny.

### SNP identification

The sequence reads were processed with Trimmomatic v0.39 [54] to remove adapters, read pairs with the percentage of N >10%, and read pairs with low-quality bases (Q ≤ 5) >50%. The cleaned reads were aligned to the pear (*P. bretschneideri*) reference genome of Dangshansuli v1.0 [11] using the Burrows–Wheeler Alignment Tool (BWA-MEM, version 0.7.16a-r118) [55]. The read pairing information and flags on the BAM (sequence alignment/map format) records were first cleaned up using SAMtools (version 1.9, http://samtools.sourceforge.net/) fixmate. The BAM records were sorted from name order into coordinate order. To reduce the number of indel miscalls in the BAM records, the raw gapped alignment was improved by using the Genome Analysis Toolkit (GATK v3.7) Realigner [56]. The Picard mark duplicates tool (http://broadinstitute.github.io/picard/) was used for masking PCR and optical duplicates. The resulting BAM file was converted to produce BCF files containing all the locations in pear genomes using the mpileup command of the bcftools program [57]. The BCF file was used for SNP and indel calling using bcftools software (v1.9) with bcftools call –vmO z parameters. We kept only bi-allelic SNP sites for further analysis to reduce the complexity. Any genotypes with supporting bases either <4 or >200 were considered low-confidence and converted to missing data. We tested the observed segregation pattern of all SNP sites against the expected Mendelian segregation in the *F*_1_ population using the *χ*^2^ test and discarded those sites with distorted segregation (*P* < .05).

### Genetic map construction and collinearity analysis

The genetic map was constructed by using the pseudo-testcross mapping strategy [58], which was described in the JoinMap software as the CP (cross pollinators) model. The CP model is used in JoinMap to perform genetic mapping in a full-sib family resulting from a cross between two individuals of an outbreeding species [30]. Since we kept only bi-allelic SNPs from the variant calling process, there were three segregation types (nnxnp, lmxll, and hkxhk) identified for further analysis. ‘lmxll’ indicates that the paternal parent was heterozygous and the maternal parent was homozygous, and the expected separation ratio of the two different genotypes of offspring (lm:ll) is 1:1. ‘nnxnp’ indicates that the paternal parent was homozygous but the maternal parent was heterozygous, and the expected genotype separation ratio (nn:np) is 1:1. ‘hkxhk’ indicates that both of the parents were heterozygous, and the expected separation ratio of three genotypes (hh:hk:kk) is 1:2:1. The hkxhk marker type serves as a bridge to merge the two parental map types. The whole genome re-sequencing strategy produced millions of high-quality SNP markers, which was beyond the computing capacity of available programs, including the widely used JoinMap (v4.1). In order to reduce the number of redundant markers, the three sets SNP markers (nnxnp, lmxll, and hkxhk) were independently loaded into Binmarker-v2.3 (https://github.com/lileiting/Binmarkers-v2) to create bins. Binmarker-v2 is a Perl script that can merge 100%-identical markers within a given genomic window (10 kb as default, 500 kb in this study) in five main steps: (i) bin markers by 10-kb window using majority rules (but if two genotypes were of equal weights, it would be treated as missing data); (ii) fill missing genotypes with majority rules; (iii) fill the breakpoints with missing genotypes (two adjacent, identically genotyped Bin markers will be filled with the missing genotype); (iv) correct miscoded genotypes with strict criteria (a genotype is different from surrounding genotypes; no missing data in surroundings genotypes; and surroundings genotypes are the same); and (v) merge 100% identical markers. A consensus map was constructed with the three sets of bin markers using the CP model. First, the maternal lh-map was constructed using the lmxll and hkxhk markers, and then the paternal nh-map was constructed using the nnxnp and hkxhk markers. Finally, the consensus genetic map LNHK-map was merged using the common markers shared by lh-map and nh-map using MergeMap software [23]. Marker order and genetic distances were obtained using the Kosambi function and regression mapping algorithm. To ensure the linkage quality of the genetic map, a series of strict standards was used in the JoinMap software. These included: exclusion from the project markers whose missing genotype are higher than 9 should be removed from the project; a higher LOD value when performing the grouping method, with a lowest independence LOD = 9 (ranging from 2 to 30, step = 1); exclusion from the project of all marker pairs showing weak linkages. The linkage group number of LNHK-map was named according to the chromosomes in the pear (*P. bretschneideri*) reference genome ‘Dangshansuli’ v1.0 (http://peargenome.njau.edu.cn) [11]. Pairwise recombination fractions were checked for all bins and visualized using R/qtl software [59].

The collinearity analysis was performed between the LNHK-map produced in this study, BD-map (‘Bayuehong’ × ‘Dangshansuli’) [2], and the ‘Dangshansuli’ genome v1.0 using a Python script by checking whether the physical position of the SNP markers was located in the bins in the LNHK-map or the scaffolds in the pear genome. If the markers in the same bins or scaffolds were mapped into the same linkage groups, they were considered as collinear. The marker-scaffold collinearity was visualized using Strudel (https://bioinf.scri.ac.uk/strudel/). Finally, ALLMAPS [60] was used to order the pear genome scaffolds based on the collinearity between the BD-map and LNHK-map linkage maps. The alignment comparison analysis of ‘Dangshansuli’-v2 and BartlettDHv2 was performed using MUMmer4 [61]. Besides, collinearity inside the LNKH-map in Table 1 was calculated as the percentage of markers that were co-anchored (the number of co-anchored markers divided by the total number of co-anchored and misaligned markers).

### Quantitative trait locus mapping analysis

QTL mapping was performed using the phenotype data of 2015 and 2016 separately with MapQTL v6.0 [29]. The non-normal phenotypic trait data were transformed using the logarithm (ln), the square root (sqrt), the cubic root (curt), and the Tukey method. The normalized traits were mapped with the interval mapping method, while any non-normal traits (even after transformation) were mapped with the Kruskal–Wallis test. For the interval mapping mapping method, QTLs with mean values higher than LOD = 2.5 [3, 17, 62] were considered significant. For the Kruskal–Wallis test, Signif. < .005 (^****^) was considered significant. The QTL intervals were defined using the 2 LOD drop-off strategy [63]. All the bin markers located on the QTL intervals in the LNHK-map were used for candidate gene identification by locating their physical position on the pear genome of ‘Dangshansuli’.

### Candidate gene selection and gene expression pattern analysis

Genes located on the physical position of bin marker of the QTL regions were used for further study. For candidate gene screening, transcriptome data were used from ‘Dangshansuli’ [11] and five other pear cultivars [64] at six key fruit development stages, including fruit set [15 days after full blooming (DAFB)], the physiological fruit drop stage (30 DAFB), the fruit rapid enlargement stage (55 DAFB), 1 month after fruit enlargement stage (85 DAFB), the pre-mature stage (115 DAFB), and the mature stage (varies by cultivar). We combined the expression pattern and phenotypic trends at different developmental stages, as previously described [65–67], to narrow down the candidate genes in QTLs. First, the RPKM (reads per kilobase per million mapped reads) values of genes in QTLs associated with each trait were selected and the cluster function of the Pheatmap/R package was used to divide these genes into different groups based on expression patterns. Groups with expression trends consistent with phenotypic developmental trends were considered candidate genes. The gene expression patterns were visualized using the Pheatmap package (https://CRAN.R-project.org/package=pheatmap) in R.

### Transient transformation of pear fruits

For the transient transformation expression analysis, we prepared a pMDC32 [68] construct to overexpress *PbrtMT1*. The full length of the coding sequence of *PbrtMT1* was PCR-amplified using Q5 (NEB) with primers PbrtMT1-F (ACGGAAGATTTCTCCTTGGGGAG) and PbrtMT1-R (GTGACCACACCATTACCAAACCAC) and then cloned into Gateway vector pCR8/GW/TOPO. After sequence confirmation, the *PbrtMT1* fragments were recombined into a pMDC32 vector, which is under the control of a cauliflower mosaic virus (CaMV) 35S promoter to create the 35S::PbrtMT1 construct. Finally, the recombinant plasmid and the control empty vector were introduced into *Agrobacterium tumefaciens* strain GV3101 by heat shock and used for transient transformation in pear fruit.

Three independent replicates of three pear fruits each (110 DAFB) from ‘Dangshansuli’ were injected (1 mL) with the construct. The transformed fruits were placed in the dark at 21°C for 24 hours, followed by 5 days under a 16/8-hour light/dark cycle at 21°C [69]. Finally, pulp samples around the injection area were collected and mixed for RNA extraction and sugar measurements.

### RNA extraction, reverse transcription, and expression profiles of genes

Total RNA was extracted using the Plant Total RNA Isolation Kit (RE-05011, ForeGene, China), and the first strand of cDNA was synthesized using TransScript^®^ First-Strand cDNA Synthesis SuperMix (AT301-02, TransGen Biotech, China). qRT–PCR was performed with 96-well plates according to the instructions of LightCycler 480 SYBR Green I Master (Roche). The PCR reactions each comprised 10 μL in total volume, including 5 μL SYBR Green I Master, 0.5 μL forward primer, 0.5 μL reverse primer, 1 μL cDNA (100 ng), and 3 μL ddH_2_O. The reaction program was 95°C for 3 minutes; 45 cycles of 95°C for 3 seconds, 60°C for 10 seconds, and 72°C for 30 seconds, with a melt curve between 60 and 95°C. Relative expression was calculated by the 2^-ΔΔCt^ method [70]. α-Tubulin (*Pbr042345.1*) was used as the internal control. All primers are listed in Supplementary Data Table S9.

## Acknowledgements

This work was financially supported by the National Key Research and Development Program (2018YFD1000200), the National Science Foundation of China (31820103012, 31725024, 31801835, 31901983), the China Agriculture Research System of MOF and MARA, and the Earmarked Fund for Jiangsu Agricultural Industry Technology System (JATS [2020]401]).

## Author contributions

M.F.Q. and L.T.L. carried out data analysis, prepared the figures, and drafted the manuscript. A.K. and J.S. provided support for data analysis, interpretation of results, and manuscript writing. M.Y.S. and M.Y.Z. helped revise the manuscript. B.B., J.Y.Z., X. Zhang, S.W.L., W.L.W., and K.J.Q. helped collect samples and collected the phenotypic data. J.P.N. and J.M.L. performed the transient transformation experiments to verify the function of *PbrtMT1*. S.L.Z. provided experimental support. J.W. designed and managed the research. M.F.Q. and L.T.L. should be considered joint first authors.

## Data availability

The datasets have been submitted to NCBI-SRA database with the BioProject ID PRJNA846875.

## Conflict of interest

The authors declare no conflict of interests.

## Supplementary data

Supplementary data is available at *Horticulture Research* online.

## Supplementary Material

supp_data_uhac141
