# Supplementary material for: Construction of a high-density bin-map and identification of fruit quality-related quantitative trait loci and functional genes in pear
Source: Hortic Res. 2022 Jun 23;9:uhac141. doi: 10.1093/hr/uhac141 (PMC9437719; doi:10.1093/hr/uhac141)
Supplement: supp_data_uhac141 [file supp_data_uhac141.zip › FigS1_BD-map_to_LNHK-map.pdf]

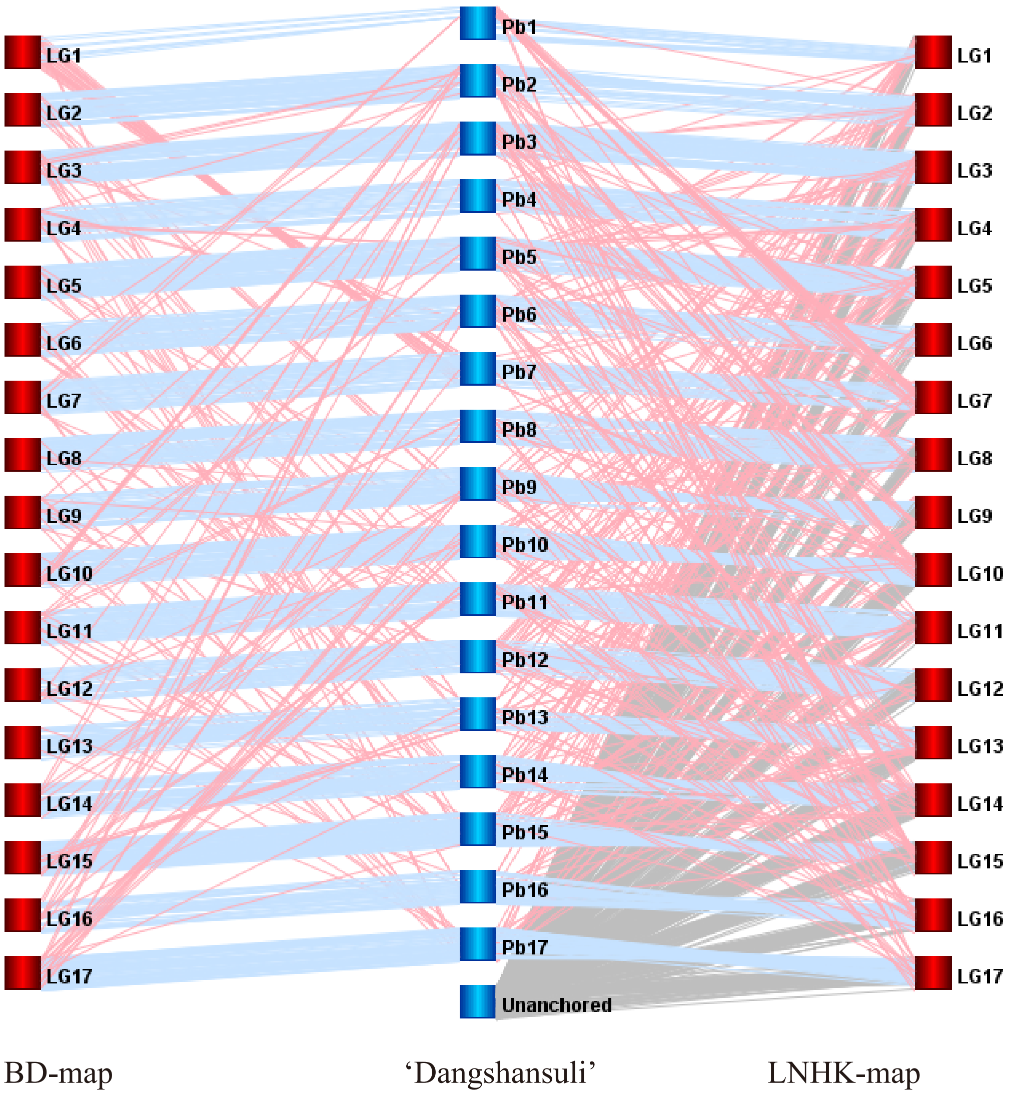

Supplementary Figure S1. Collinearity analysis of 'LNHK-map', BD-map and the 'Dangshan-suli' genome. Blue lines represent markers that originate from the same scaffold and are mapped in the same linkage group, while the red lines represent markers that originate from the same scaffold but are mapped to different linkage groups. Gray lines represent unassembled scaffolds that are mapped on the 'LNHK-map'
