# Supplementary material for: Construction of a high-density bin-map and identification of fruit quality-related quantitative trait loci and functional genes in pear
Source: Hortic Res. 2022 Jun 23;9:uhac141. doi: 10.1093/hr/uhac141 (PMC9437719; doi:10.1093/hr/uhac141)
Supplement: supp_data_uhac141 [file supp_data_uhac141.zip › FigS3_PbrLCA2.pdf]

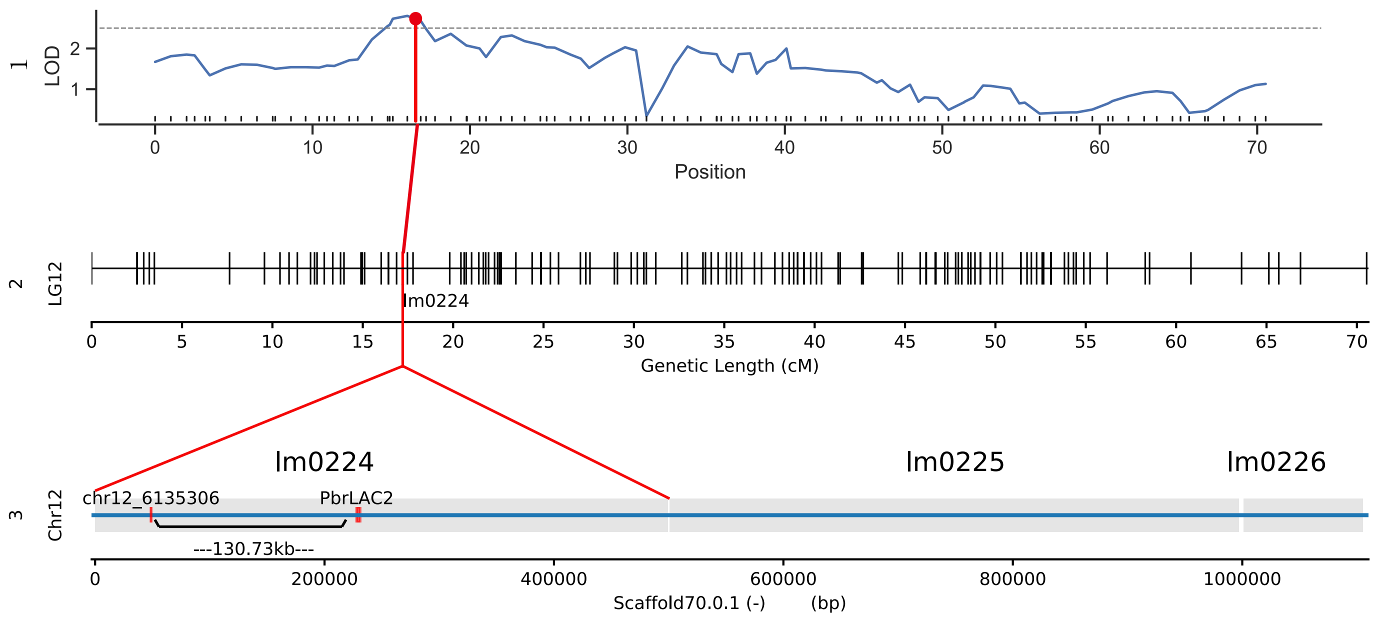

Supplementary Figure S3. Genetic and physical position of stone cell content related candidate gene *PbrLAC2*. The top shows the result of QTL mapping using IM method. The black dash line is LOD threshold value (LOD = 2.5) to identify potential QTLs. The red dot in LG12 is a significant associated SNP in single marker analysis. The middle shows a detailed genetic position of overlapped marker 'lm0224' in LG12. The bottom shows a physical position of identified SNP (red bar), binmarker lm0224 (gray shadow), and candidate gene (red bar).
