# Supplementary material for: Construction of a high-density bin-map and identification of fruit quality-related quantitative trait loci and functional genes in pear
Source: Hortic Res. 2022 Jun 23;9:uhac141. doi: 10.1093/hr/uhac141 (PMC9437719; doi:10.1093/hr/uhac141)
Supplement: supp_data_uhac141 [file supp_data_uhac141.zip › FigS4.pdf]

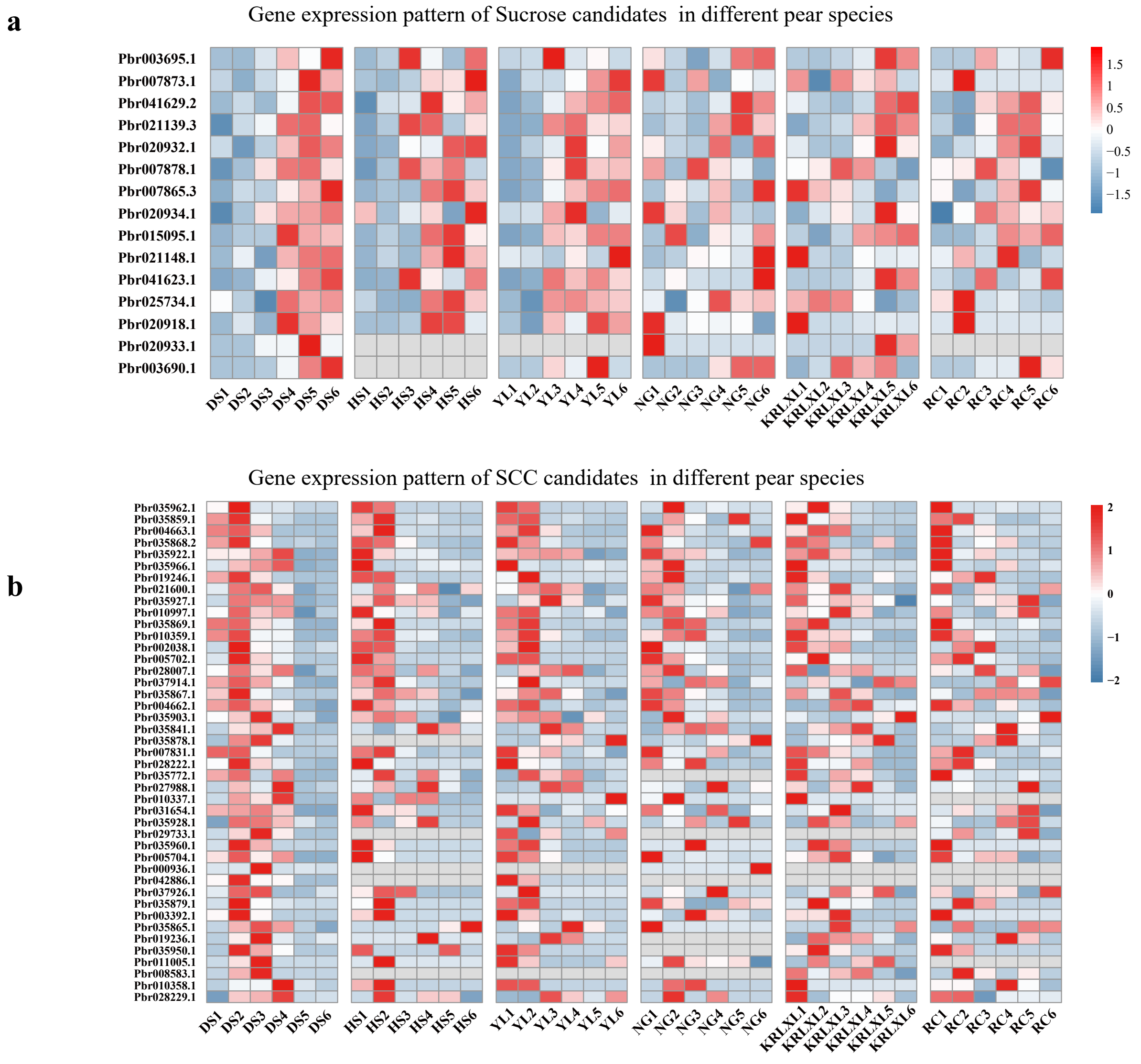

Suppplementary Figure S4. a: Gene expression pattern of sucrose candidates in different pear species. **b**: Gene expression pattern of stone cell content in different pear species. ‘DS’, ‘HS’, ‘YL’, ’NG’, ‘KRLXL’, ‘RC’ represent the main cultivar from different pear species, ‘Dangshansuli’, ‘Hosui’, ‘Yali’, ‘Nanguoli’, ‘Kuerlexiangli’ and ‘Starkrimson’. 1 to 6 represent different developmental stages: ‘Setting stage’, ‘Physiological fruit dropping stage’, ‘Rapid expansion stage’, ‘One week after rapid expansion stage’, ‘Pre-mature stage’ and ‘Mature stage’, respectively. The expression data were normalized by R package *Pheatmap scale = ‘row’* method.
