# Supplementary figures and images for: Construction of a high-density bin-map and identification of fruit quality-related quantitative trait loci and functional genes in pear
Source: Hortic Res. 2022 Jun 23;9:uhac141. doi: 10.1093/hr/uhac141 (PMC9437719; doi:10.1093/hr/uhac141)

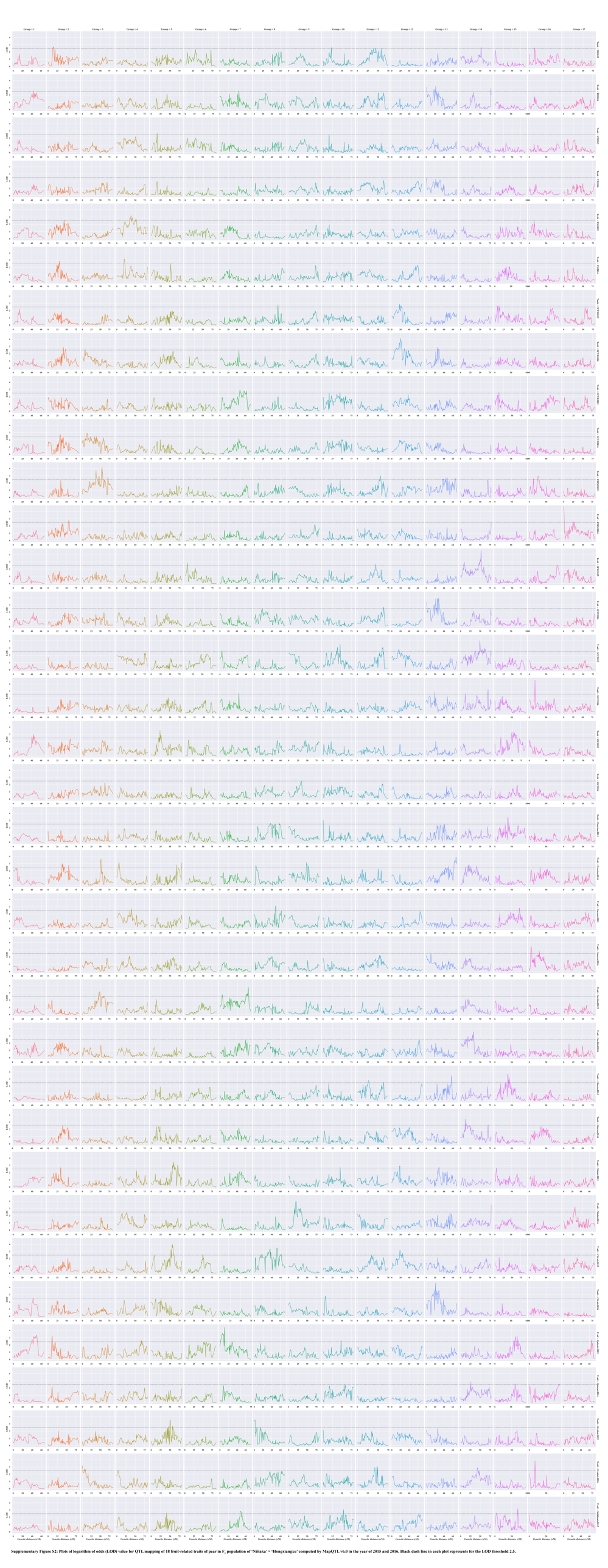

Supplement: supp_data_uhac141 [file supp_data_uhac141.zip › FigS2_LOD_Value.pdf]
